# Invisible Intelligences

**Published:** 1888-08

**Authors:** 


### INVISIBLE INTELLIGENCES.

"The evidence of invisible intelligences is so cumulatively strong as to put the matter beyond doubt. The basic facts of the Christian religion rest entirely on the spirit resurrection of the Christ and His appearance to His disciples and His ascent into another world. The whole bible would go to pieces but for similar facts from Genesis to Revelation. In their day the prophets who saw and heard supernaturally, as it is called, were regarded as insane. No one will be bold enough to charge Professor

Winchell with insanity. He says: 'The unseen world is destined to became like a newly-discovered continent. We shall visit it, we shall hold communion with it, we shall wonder how so many thousands of years could have been passed without our being introduced to it. We shall learn of other modes of existence, intermediate, perhaps, between body and spirit, having the forms and limitations of space peculiar to matter, with the penetrability and invisibility of spirit.' But the foregoing is but a trifle of what he says in one of his scientific works. Out with a strait jacket! But wait a moment. It will better fit the wiseacres who would limit the capacities of the human soul. To them everything not sensual is 'impossible.' Prof. Winchell sees the onward sweep of occult science, and, as a proof the works of Jacob Behman, written two hundred years ago, are about coming into the market, for there is a demand for them. Emerson did not laugh at Behman, nor at the Swedish seer, nor at the old philosophical speculator of Konigsberg." —*New York Sunday Mercury.*

